# Supplementary material for: Mesoscale DNA features impact APOBEC3A and APOBEC3B deaminase activity and shape tumor mutational landscapes
Source: Nat Commun. 2024 Mar 18;15:2370. doi: 10.1038/s41467-024-45909-5 (PMC10948877; doi:10.1038/s41467-024-45909-5)
Supplement: Supplementary file 1 — Supplementary Information [file 41467_2024_45909_MOESM1_ESM.pdf]

Supplementary information for

Mesoscale DNA Features Impact APOBEC3A and APOBEC3B Deaminase Activity and Shape Tumor Mutational Landscapes

Ambrocio Sanchez, Pedro Ortega, Ramin Sakhtemani, Lavanya Manjunath,

Sunwoo Oh, Elodie Bournique, Alexandrea Becker, Kyumin Kim, Cameron Durfee,

Nuri Alpay Temiz, Xiaojiang S. Chen, Reuben S. Harris, Michael S. Lawrence

& Rémi Buisson

Supplementary Figure 1

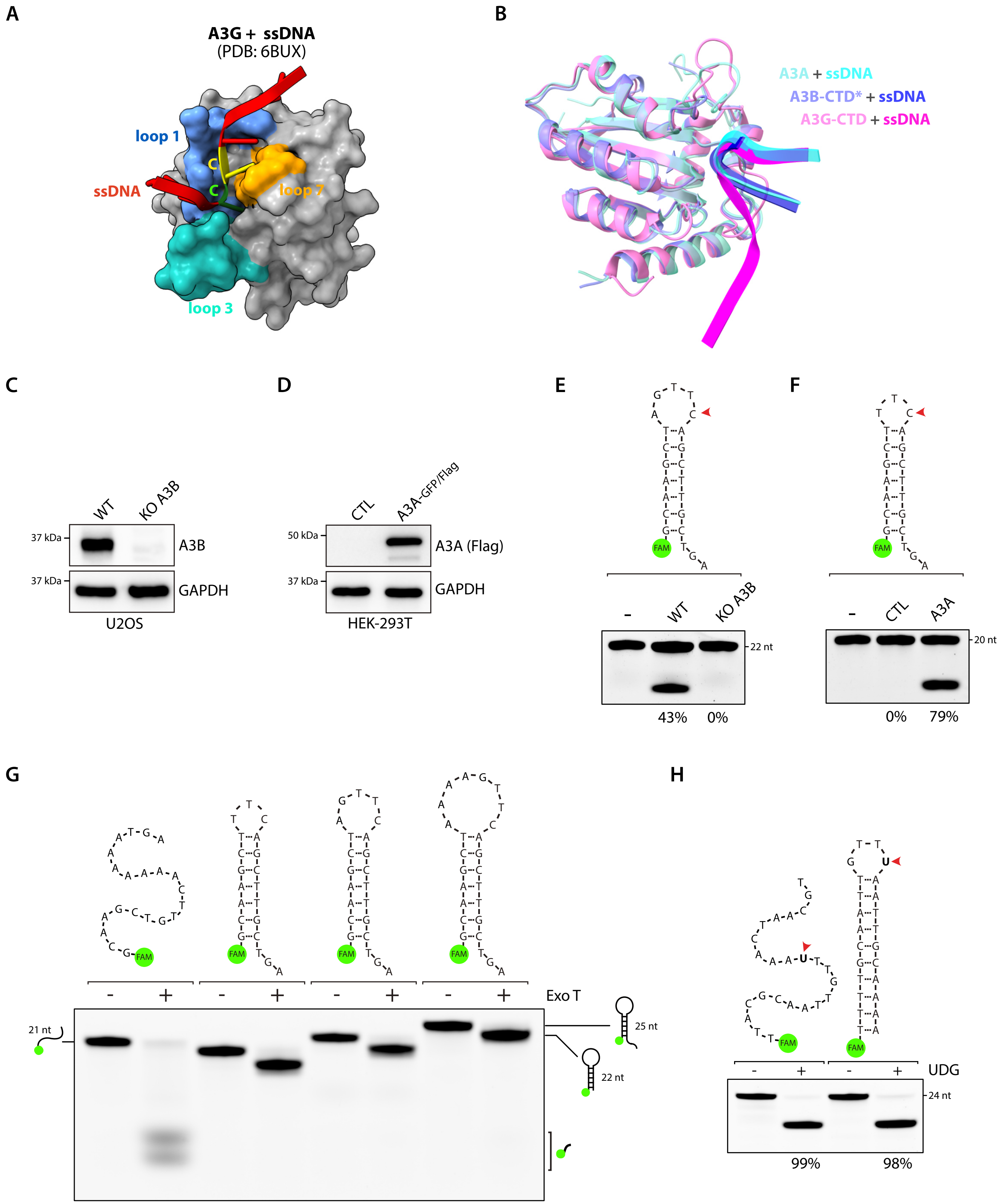

Supplementary Figure 1. A. Molecular surface of the A3G-CTD CTD in complex with ssDNA. The loops surrounding the active site have been color coded as shown. PDB IDs shown for reference. **B.** Superposition of A3A, A3B-CTD, and A3G-CTD structures in complex with ssDNA. The asterisk (*) indicates that certain amino acid sequences of A3B were modified. **C.** U2OS cells wild type or knockout for A3B were analyzed by western blot with the indicated antibodies. **D.** Wild-type or A3A-expressing HEK-293T cells were analyzed by western blot with the indicated antibodies. **E.** A3B deamination activity assay using 20 µg of U2OS whole cell extract of wild type or A3B knockout cells on indicated DNA stem-loop oligonucleotide. The percentage of cleavage is indicated. **F.** A3A deamination activity assay was performed with 1 µg of whole cell extract of HEK-293T wild type cells or with HEK-293T cells expressing A3A on indicated DNA stem-loop oligonucleotide. The percentage of cleavage is indicated. **G.** Indicated DNA oligonucleotides were incubated with Exonuclease T and the DNA degradation for each substrate was monitored on a 20% denaturing PAGE gel. **H.** Both a single-stranded DNA and a hairpin substrate with a TpU site were incubated with reaction buffer in the presence or in the absence of recombinant UDG (0.008 U). Source data are provided as a Source Data file.

Supplementary Figure 2

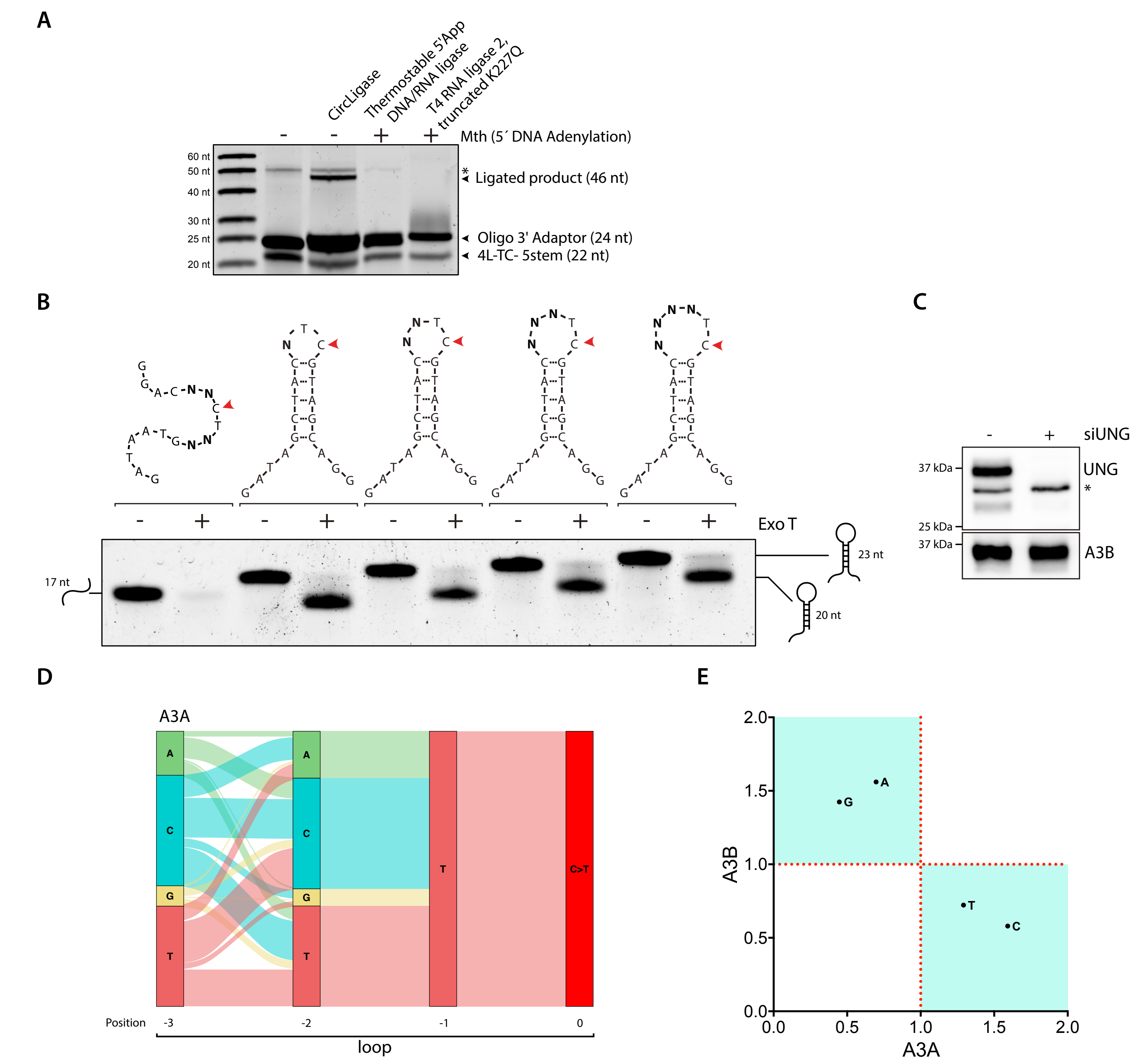

Supplementary Figure 2. A. Indicated ligases were tested to identify which one promotes efficient ligation of Oligo 3' Adaptor with 4L-TC-5stem oligo. Two of the three ligases required a 5' pre-adenylation step, which was performed using Mth ligase. Only the CircLigase proved to be an efficient single strand DNA ligase as indicated by the presence of a 46 nt band. **B.** Indicated DNA oligonucleotides used during Oligo-seq experiments, were incubated with Exonuclease T and the DNA degradation for each substrate was monitored on a 20% denaturing PAGE gel. **C.** U2OS cells wild type or knocked down for UNG1/2 were analyzed by western blot with the indicated antibodies. Asterisk (*) indicates a nonspecific band. **D.** A river plot depicting the relative frequency of each nucleotide at the indicated position of a 4-nt hairpin loop and its association with the nucleotides located before or after. **E.** Scatter plot of the fold enrichment and depletion for each of the 4 DNA bases possibly present in the 3-nt hairpin loop shown in **Figure 2B**. Source data are provided as a Source Data file.

Supplementary Figure 3

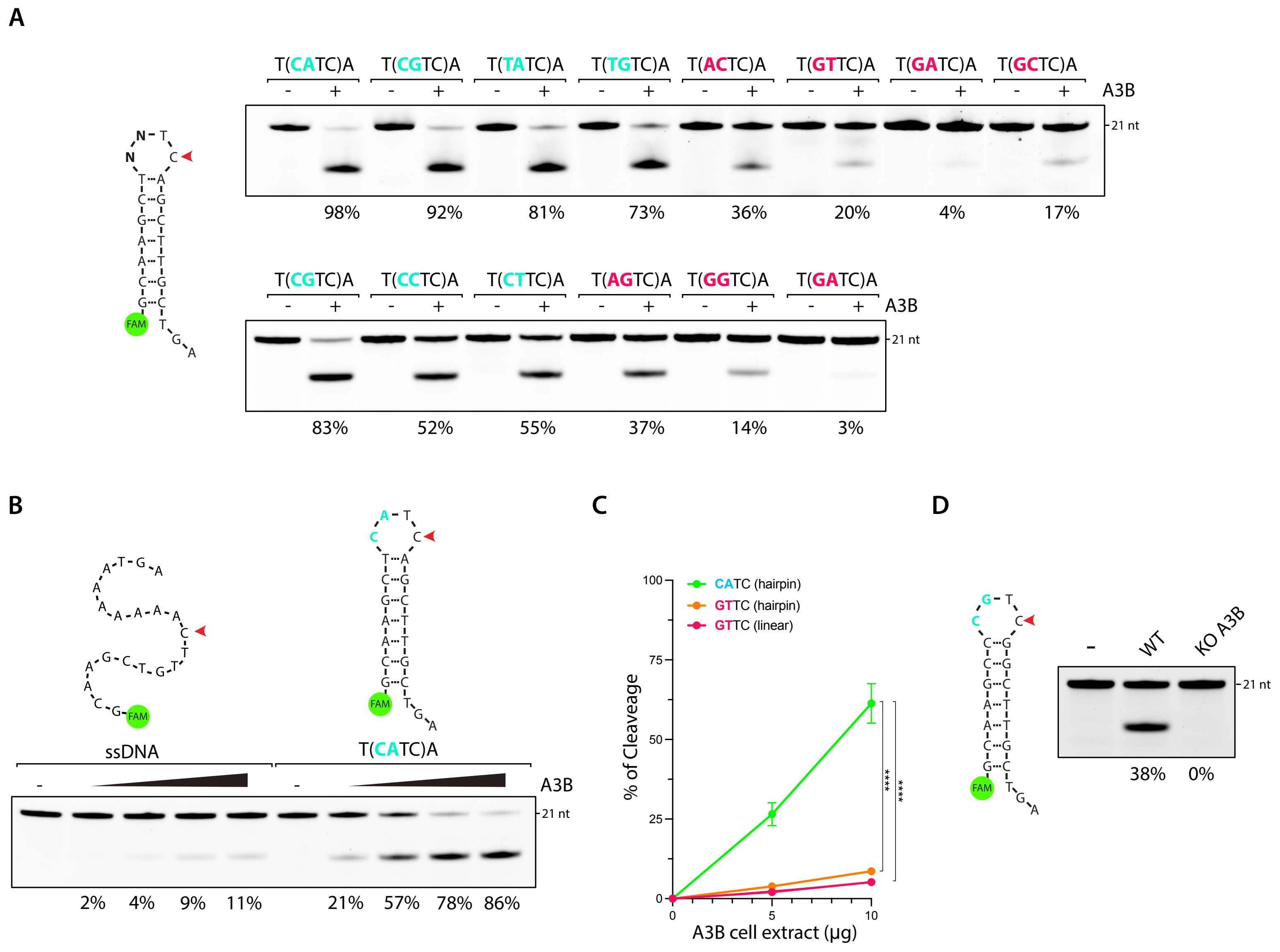

Supplementary Figure 3. A. A3B deamination activity assay using 30 μg of U2OS whole cell extract on indicated DNA oligonucleotides. The loop sequences are shown enclosed within parentheses. The percentage of cleavage is indicated. **B.** A3B deamination activity assay using 5 to 30 μg of U2OS whole cell extract on indicated DNA oligonucleotides. The loop sequences are shown enclosed within parentheses. The percentage of cleavage is indicated. **C.** Quantification of the A3B deamination activity on the indicated DNA substrates. Data are presented as mean values +/- S.D. (Number of biological replicates, n = 4; and ****p<0.0001 [two-tailed unpaired t-test]). **D.** A3B deamination activity assay using 30 μg of U2OS whole cell extract of wild type or knockout for A3B cells on indicated DNA stem-loop oligonucleotides. The loop sequences are shown enclosed within parentheses. The percentage of cleavage is indicated. Source data are provided as a Source Data file.

Supplementary Figure 4

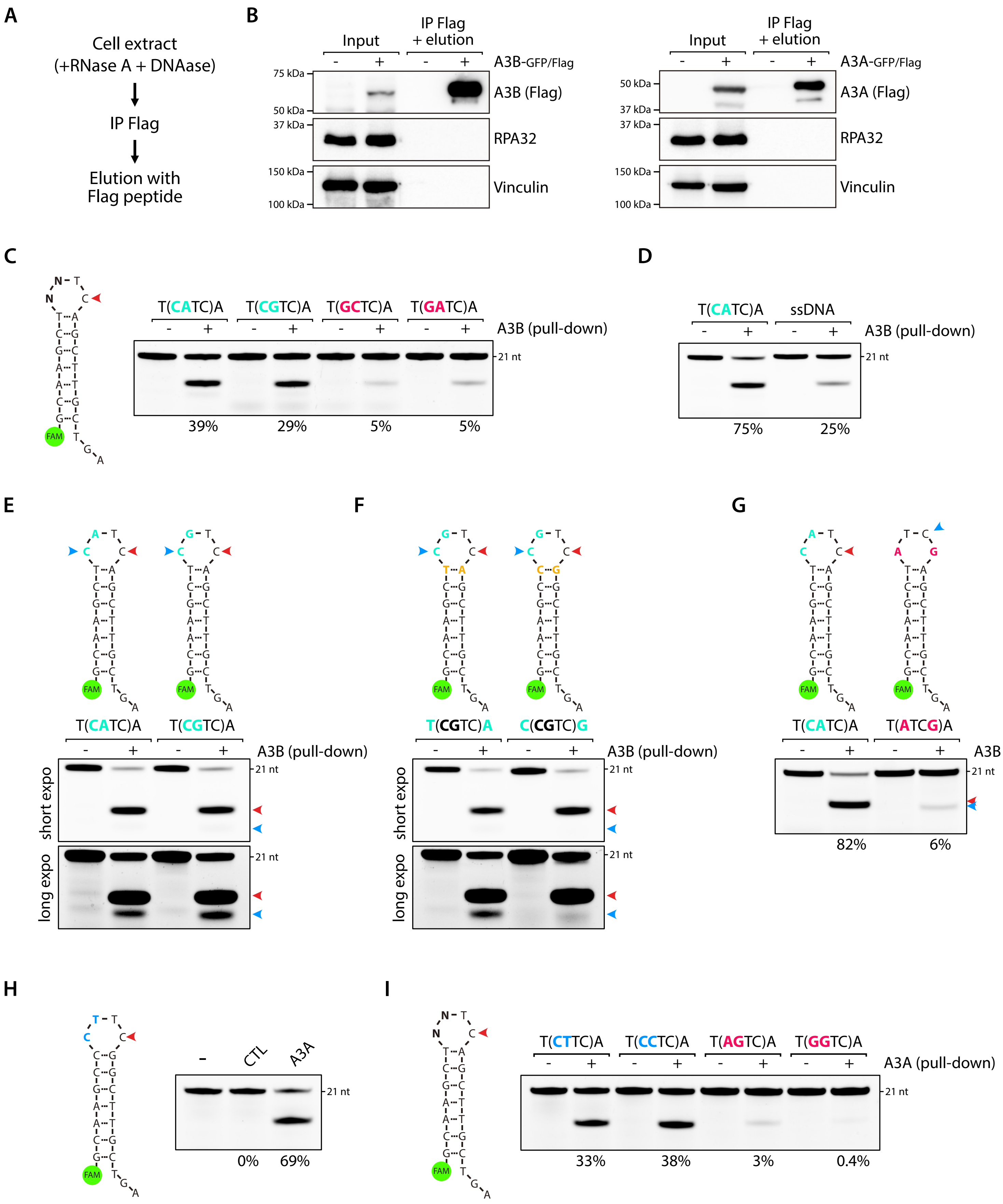

Supplementary Figure 4. A. A3B pulldown purification strategy. **B.** Flag pulldown of A3A-GFP/Flag or A3B-GFP/Flag expressed in HEK-293T cells were analyzed by western blot with the indicated antibodies. **C-F.** A3B deamination activity was performed with purified A3B by immunoprecipitation of U2OS cells stably expressing flag-tagged A3B on the indicated substrates. The loop sequences are shown enclosed within parentheses. The percentage of cleavage is indicated. **G.** A3B deamination activity assay using 20 µg of U2OS whole cell extract expressing endogenous A3B on indicated DNA stem-loop oligonucleotides. The loop sequences are shown enclosed within parentheses. The percentage of cleavage is indicated. **H.** A3A deamination activity assay was performed with 5 µg of HEK-293T whole cell extract of wild-type cells or cells expressing A3A on indicated DNA stem-loop oligonucleotides. **I.** Deamination activity assay was performed using purified A3A by immunoprecipitation from HEK-293T cells on indicated DNA stem-loop oligonucleotides. The loop sequences are shown enclosed within parentheses. The percentage of cleavage is indicated. Source data are provided as a Source Data file.

Supplementary Figure 5

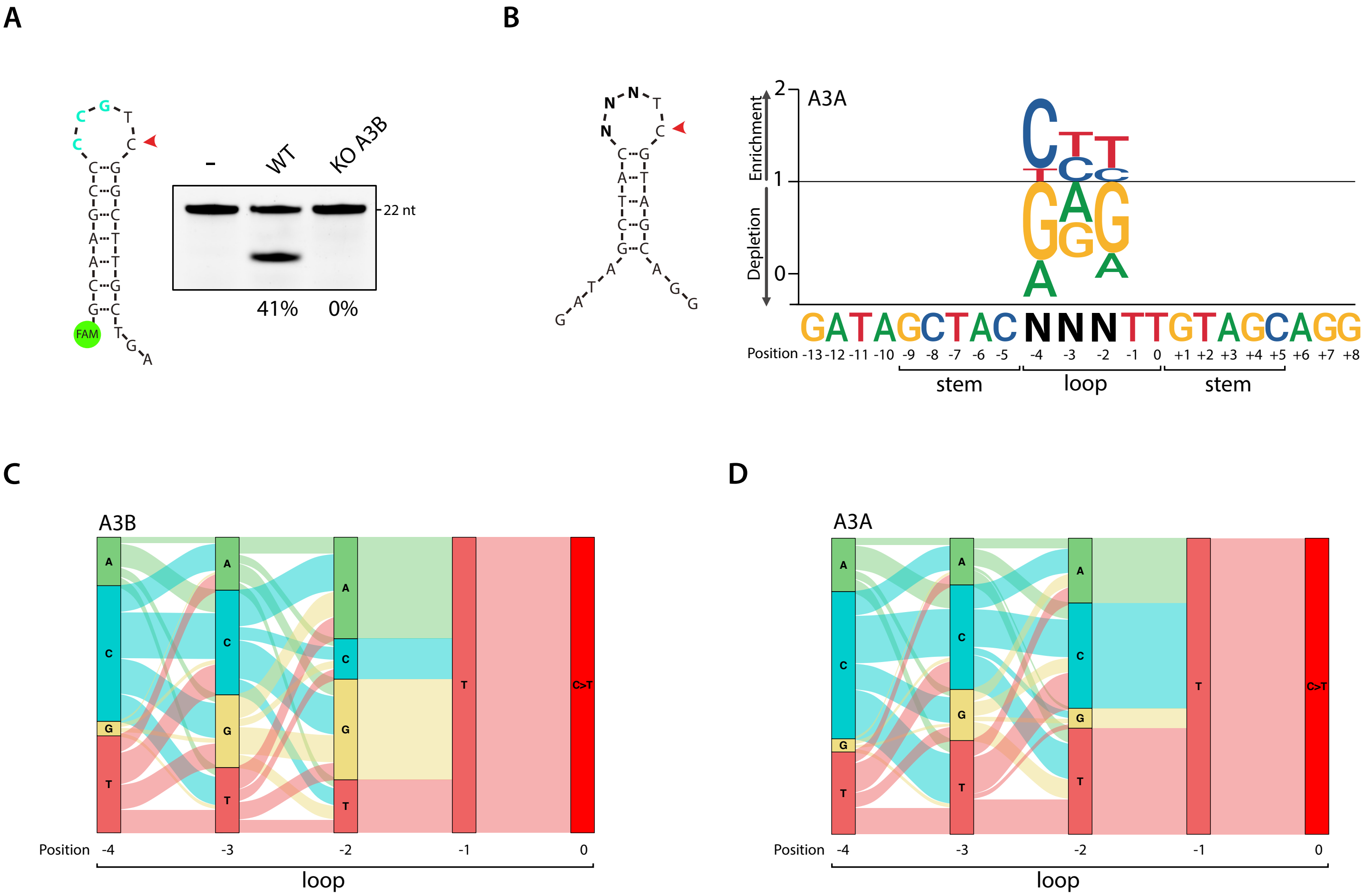

Supplementary Figure 5. A. A3B deamination activity was performed with 20 µg of U2OS whole cell extract of wild type or A3B knockout cells on indicated DNA stem-loop oligonucleotide. The percentage of cleavage is indicated. **B.** A sequence logogram showing the fold enrichment and depletion for each of the four DNA bases at the -2, -3, and -4 position after deamination of the cytosine by A3A. **C-D.** A river plot depicting the relative frequency of each nucleotide at the indicated position of a 5-nt hairpin loop and its association with the nucleotides located before or after. Source data are provided as a Source Data file.

Supplementary Figure 6

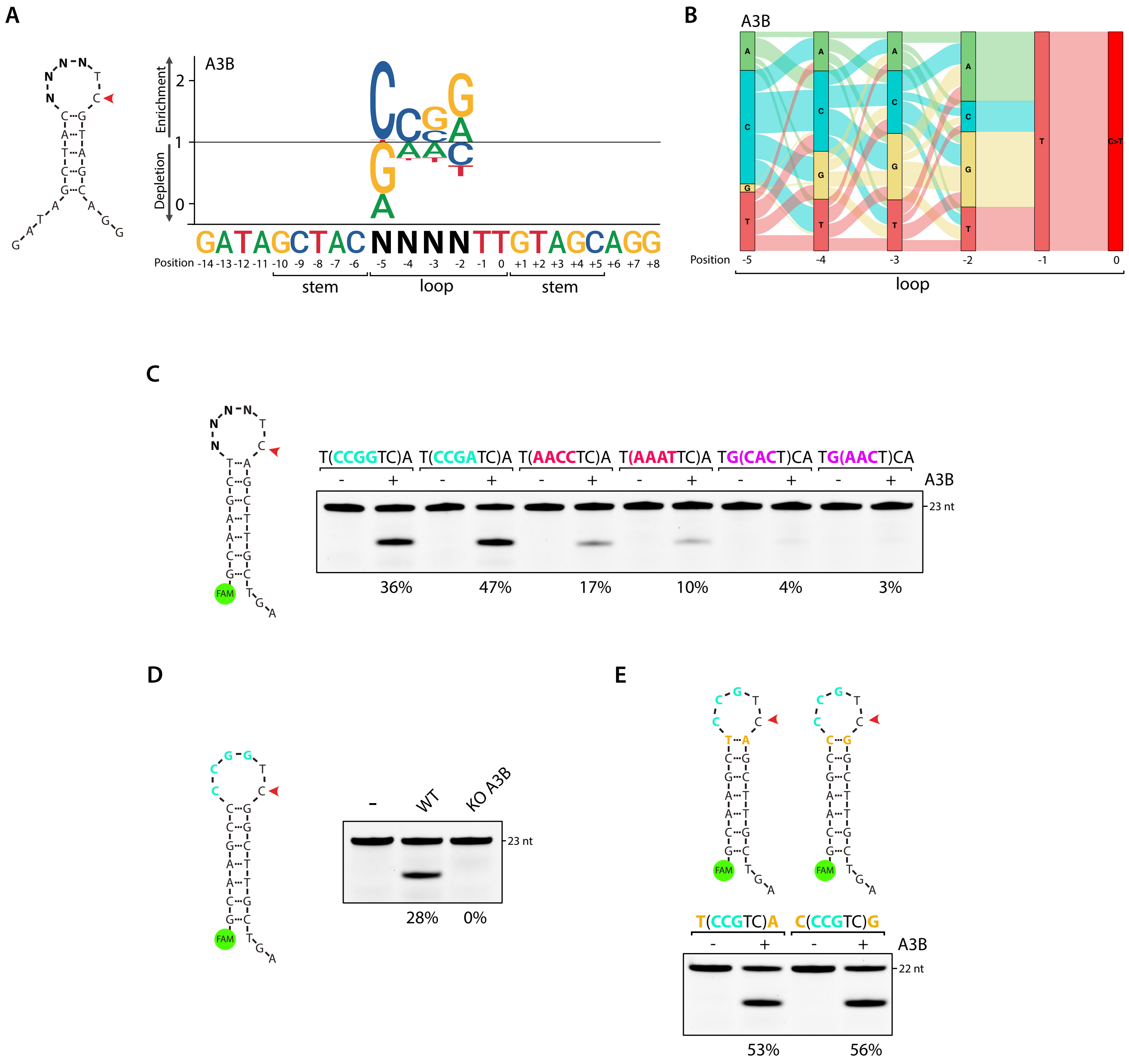

Supplementary Figure 6. A. Sequence logogram showing the fold enrichment and depletion for each of the four DNA bases at the -2, -3, -4, and -5 position after deamination of the cytosine by A3B. **B.** A river plot depicting the relative frequency of each nucleotide at the indicated position of a 6-nt hairpin loop and its association with the nucleotides located before or after. **C–E.** A3B deamination activity assay was performed with 20 µg of U2OS whole cell extract of wild type or A3B knockout cells on indicated DNA stem-loop oligonucleotides. The loop sequences are shown enclosed within parentheses. The percentage of cleavage is indicated. Source data are provided as a Source Data file.

Supplementary Figure 7

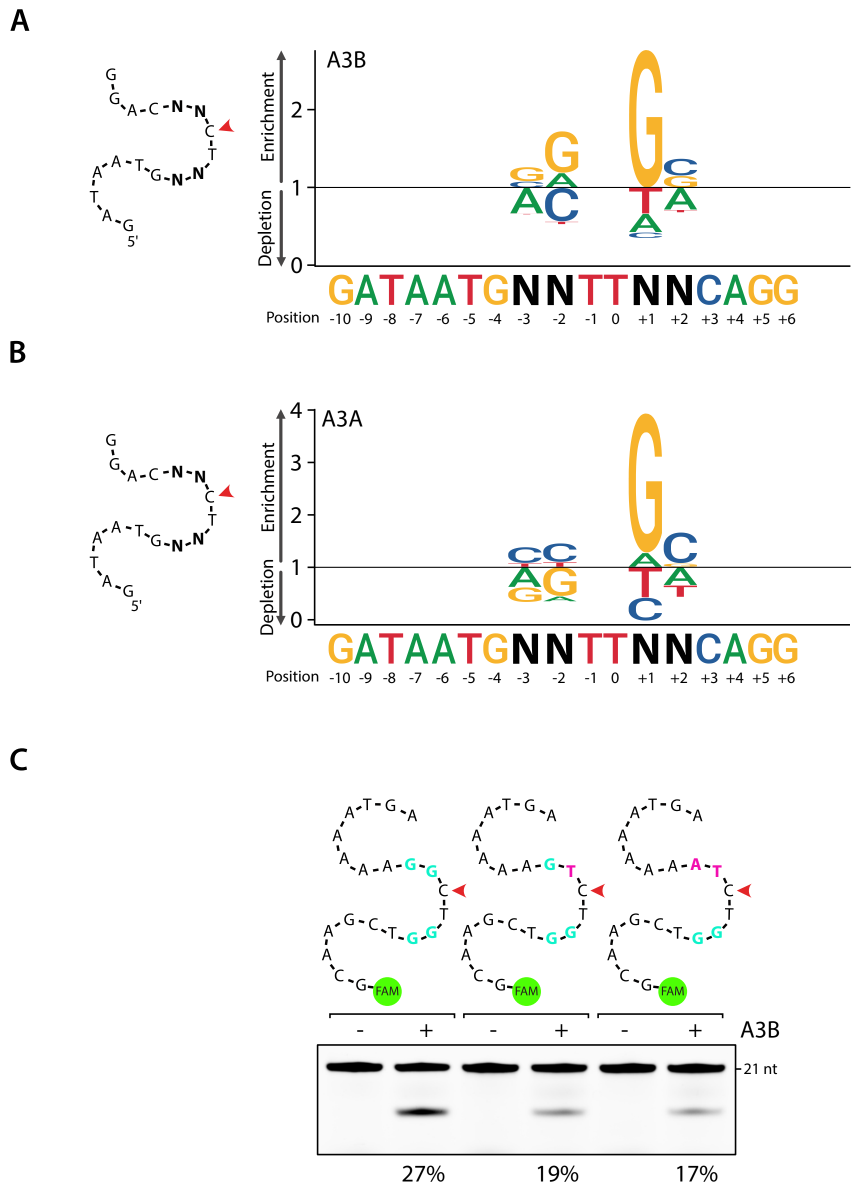

Supplementary Figure 7. A–B. Sequence logograms showing the fold enrichment and depletion for each of the four DNA bases at the -3, -2, +1 and +2 positions after deamination of the cytosine by A3B (**A**) and A3A (**B**). **C.** A3B deamination activity assay was performed with 20 µg of U2OS whole cell extract on indicated single-stranded DNA oligonucleotides. The percentage of cleavage is indicated. Source data are provided as a Source Data file.

Supplementary Figure 8

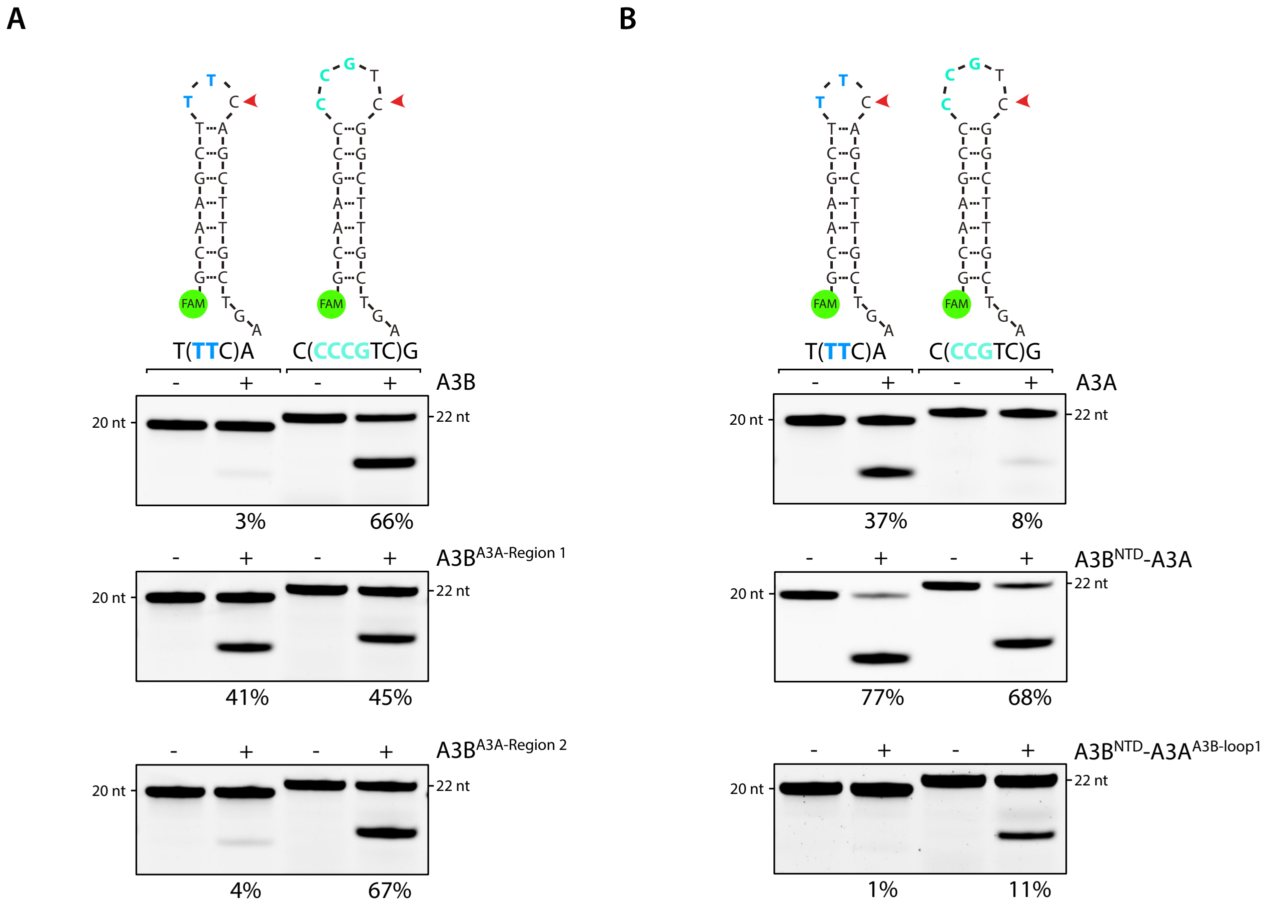

Supplementary Figure 8. A-B. Deamination activity assay was performed with HEK-293T whole cell extract expressing indicated constructs on 3-nt and 5-nt DNA stem-loop oligonucleotides preferentially targeted by A3A and A3B respectively. The loop sequences are shown enclosed within parentheses. The percentage of cleavage is indicated. Source data are provided as a Source Data file.

Supplementary Figure 9

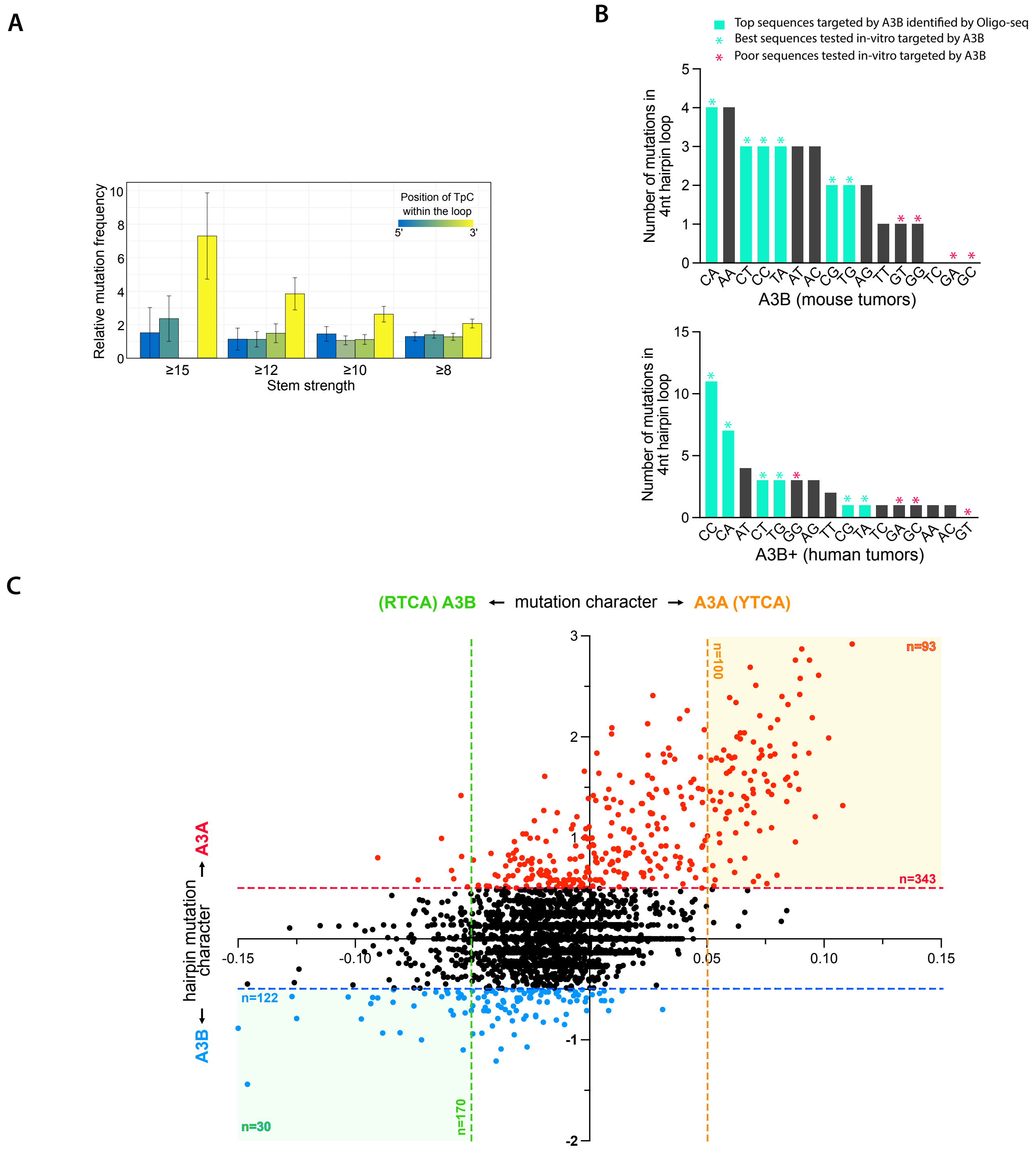

Supplementary Figure 9. A. Relative mutation frequency in A3B tumor samples. Mutation levels were classified in terms of the strength of hairpin base-pairing potential (x-axis) and by their position in the hairpin loop (color gradient). Error bars represent 95% confidence intervals. **B.** Bar graph showing the number of mutations in 4 nt hairpin loop found in mouse tumors (**top**) or human tumors (**bottom**) expressing A3B deconvolved by their dinucleotide sequences preceding the TpC sites. Dinucleotide sequences that were cross-examined with data obtained from Oligo-seq and with the *in vitro* deaminase assay are indicated by the turquoise color and asterisk (*), respectively. **C.** Patient tumor samples described in **Figure 6C** were plotted in function of their mutation levels in RTCA or YTCA motifs versus the levels of hairpin mutation character. Indicated thresholds were applied to identify the number of patient tumors that accumulated high levels of YTCA mutations (100 patient tumors), RTCA mutations (70 patient tumors), A3A hairpin mutation character (343 patient tumors), or A3B hairpin mutation character (122 patient tumors). The square box highlights patient tumors identified by both methods. Source data are provided as a Source Data file.

SUPPLEMENTARY METHODS

Oligonucleotides

List of oligonucleotide sequences used in the *in vitro* DNA deaminase assay for this study.

OLIGO	SEQUENCE
NUP93-3L	5'-(6-FAM)-GCAAGCTTTCAGCTTGCTGA
NUP93-4L	5'-(6-FAM)-GCAAGCTGTTCAGCTTGCTGA
NUP93-5L	5'-(6-FAM)-GCAAGCTAGTTCAGCTTGCTGA
NUP93-6L	5'-(6-FAM)-GCAAGCTAAGTTCAGCTTGCTGA
NUP93-7L	5'-(6-FAM)-GCAAGCTAAAGTTCAGCTTGCTGA
NUP93-8L	5'-(6-FAM)-GCAAGCTAAAAGTTCAGCTTGCTGA
NUP93-CATC	5'-(6-FAM)-GCAAGCTCATCAGCTTGCTGA
NUP93-CGTC	5'-(6-FAM)-GCAAGCTCGTCAGCTTGCTGA
NUP93-GATC	5'-(6-FAM)-GCAAGCTGATCAGCTTGCTGA
NUP93-GCTC	5’-(6-FAM)-GCAAGCTGCTCAGCTTGCTGA
NUP93-CTTC	5’-(6-FAM)-GCAAGCTCTTCAGCTTGCTGA
NUP93-CCTC	5’-(6-FAM)-GCAAGCTCCTCAGCTTGCTGA
NUP93-GGTC	5'-(6-FAM)-GCAAGCTGGTCAGCTTGCTGA
NUP93-AGTC	5'-(6-FAM)-GCAAGCTAGTCAGCTTGCTGA
NUP93-5L-CCG	5’-(6-FAM)-GCAAGCTCCGTCAGCTTGCTGA
NUP93-5L-CCA	5’-(6-FAM)-GCAAGCTCCATCAGCTTGCTGA
NUP93-5L-GAC	5’-(6-FAM)-GCAAGCTGACTCAGCTTGCTGA
NUP93-5L-GCC	5’-(6-FAM)-GCAAGCTGCCTCAGCTTGCTGA
NUP93-5L-AAT	5’-(6-FAM)-GCAAGCTAATTCAGCTTGCTGA
NUP93-5L-AAC	5’-(6-FAM)-GCAAGCTAACTCAGCTTGCTGA
NUP93-3L-C-ATC-G	5’-(6-FAM)-GCAAGCCATCGGCTTGCTGA
NUP93-C-CGTC-G	5'-(6-FAM)-GCAAGCCCGTCGGCTTGCTGA
NUP93-5L-C-CCG-G	5’-(6-FAM)-GCAAGCCCCGTCGGCTTGCTGA
NUP93-6L-C-CCGG-G	5’-(6-FAM)-GCAAGCCCCGGTCGGCTTGCTGA
NUP93-6L-T-CCGG-A	5’-(6-FAM)-GCAAGCTCCGGTCAGCTTGCTGA
NUP93-6L-T-CCGA-A	5’-(6-FAM)-GCAAGCTCCGATCAGCTTGCTGA
NUP93-6L-T-AACC-A	5’-(6-FAM)-GCAAGCTAACCTCAGCTTGCTGA
NUP93-6L-T-AAAT-A	5’-(6-FAM)-GCAAGCTAAATTCAGCTTGCTGA
NUP93-6L-T-GCAC-A	5’-(6-FAM)-GCAAGCTGCACTCAGCTTGCTGA
NUP93-6L-T-GAAC-A	5’-(6-FAM)-GCAAGCTGAACTCAGCTTGCTGA
ssDNA-GG-GG	5’-(6-FAM)-GCAAGCTGGTCGGAAAAATGA
PLEKHS1-GTTU	5’-(6-FAM)-TTTTGCAATTGTTUAATTGCAAAA
ssDNA-GTTU	5’-(6-FAM)-TTACGCAATTGTTUAAACTAACGT
NUP93-TATC	5’-(6-FAM)-GCAAGCTTATCAGCTTGCTGA
NUP93-A3B-4L-#1 (TGTC)	5'-(6-FAM)-GCAAGCTTGTCAGCTTGCTGA
NUP93-ACTC	5’-(6-FAM)-GCAAGCTACTCAGCTTGCTGA
NUP93-GTTC	5’-(6-FAM)-GCAAGCTGTTCAGCTTGCTGA
NUP93-GATC	5’-(6-FAM)-GCAAGCTGATCAGCTTGCTGA
NUP93-GCTC	5’-(6-FAM)-GCAAGCTGCTCAGCTTGCTGA
NUP93-4L-T(ATCG)A	5’-(6-FAM)-GCAAGCTATCGAGCTTGCTGA
ssDNA-GG-TG	5’-(6-FAM)-GCAAGCTGGTCTGAAAAATGA
ssDNA-GG-TA	5’-(6-FAM)-GCAAGCTGGTCTAAAAAATGA
ssDNA	5'-(6-FAM)-GCAAGCTGTTCAAAAAAATGA

List of oligonucleotide sequences used in the Oligo-seq assay for this study.

OLIGO	SEQUENCE
Oligo-3' adaptor	5'-/Phos/AGATCGGAAGAGCACACGTCTGAA/3ddC/3'
Oligo-Reverse primer	5'-/Phos/AGATCGGAAGAGCGTCGTGTAGGGAAAGAG/iSp18/GTGACTGGAGTTCAGACGTGTGCTC-3'
Fwd Primer	5'-AATGATACGGCGACCACCGAGATCTACACTCTTTCCCTACACGACGCTC-3'
Rev Primer Index_1	5'-CAAGCAGAAGACGGCATACGAGATCGTGATGTGACTGGAGTTCAGACGGTGTGCTCTTCCGATCT-3'
Rev Primer Index_2	5'-CAAGCAGAAGACGGCATACGAGATACATCGGTGACTGGAGTTCAGACGGTGTGCTCTTCCGATCT-3'
Rev Primer Index_3	5'-CAAGCAGAAGACGGCATACGAGATGCCTAAGTGACTGGAGTTCAGACGGTGTGCTCTTCCGATCT-3'
Rev Primer Index_4	5'-CAAGCAGAAGACGGCATACGAGATTGGTCAGTGACTGGAGTTCAGACGGTGTGCTCTTCCGATCT-3'
Rev Primer Index_5	5'-CAAGCAGAAGACGGCATACGAGATCACTGTGTGACTGGAGTTCAGACGGTGTGCTCTTCCGATCT-3'
Rev Primer Index_6	5'-CAAGCAGAAGACGGCATACGAGATATTGGCGTGACTGGAGTTCAGACGGTGTGCTCTTCCGATCT-3'
Rev Primer Index_7	5'-CAAGCAGAAGACGGCATACGAGATGATCTGGTGACTGGAGTTCAGACGGTGTGCTCTTCCGATCT-3'
Rev Primer Index_8	5'-CAAGCAGAAGACGGCATACGAGATTCAAGTGTGACTGGAGTTCAGACGGTGTGCTCTTCCGATCT-3'
Rev Primer Index_9	5'-CAAGCAGAAGACGGCATACGAGATCTGATCGTGACTGGAGTTCAGACGGTGTGCTCTTCCGATCT-3'
Rev Primer Index_10	5'-CAAGCAGAAGACGGCATACGAGATAAGCTAGTGACTGGAGTTCAGACGGTGTGCTCTTCCGATCT-3'
Rev Primer Index_11	5'-CAAGCAGAAGACGGCATACGAGATGTAGCCGTGACTGGAGTTCAGACGGTGTGCTCTTCCGATCT-3'
Rev Primer Index_12	5'-CAAGCAGAAGACGGCATACGAGATTACAAGGTGACTGGAGTTCAGACGGTGTGCTCTTCCGATCT-3'
3L-TC-5stem	5'-GATAGCTACNTCGTAGCAGG-3'
4L-TC-5stem	5'-GATAGCTACNNTCGTAGCAGG-3'
5L-TC-5stem	5'-GATAGCTACNNNTCGTAGCAGG-3'
6L-TC-5stem	5'-GATAGCTACNNNNTCGTAGCAGG-3'
4loop-ss-TC	5'-GATAATGNNTCNNCAGG-3'

Bioinformatic analyses of mouse and human tumors

Mutation calls from human whole-genome sequencing (WGS) were obtained from TCGA and the International Cancer Genome Consortium Pan-Cancer Analysis of Whole Genomes project, and other projects (1, 2), as described below. Mutation calls from mouse whole-genome sequencing (WGS) and whole exome sequencing (WES) were obtained from mouse tumors driven by A3A or A3B expression previously published (3, 4). We restricted our analysis to somatic single-nucleotide variants (SSNVs) and excluded patients with fewer than 500 SSNVs in the genome, yielding a final WGS dataset comprising 2644 unique patients spanning 35 tumor types. Mutations were analyzed by the analyze_mutations function of ApoHP. Mutation signatures were analyzed by NMF, using $k = 8$, revealing a set of signatures corresponding to known mutational processes, including APOBEC enzyme activity. Each patient was characterized by *frac_apobec*, the fraction

of its mutations assigned to the APOBEC signature. Cohorts of APOBEC+ patients were defined by imposing a threshold on *frac_apobec*. Our initial analyses used a set of 110 patients with *frac_apobec* ≥ 50%. We repeated some analyses using a cutoff of 10% or 90%, with similar results. To minimize the influence of potentially overlapping hypermutation processes such as MSI, Smoking, UV, POLE, or ESO, we excluded "MSUPE+" patients, those with at least 10% of their mutations assigned by NMF to one of these five other hypermutation processes.

To measure the joint influence of hairpin-forming potential and local sequence context on the relative mutation frequency of APOBEC signature mutations, we binned genomic cytosines into classes of equivalent cytosines that share hairpin characteristics and local sequence features. For each class of genomic cytosines, we counted the number of sites (N) in the genome that belong to this class, as well as the number of mutations (n) observed in the sample cohort being analyzed. We then calculated the ratio (n/N), which represents the number of mutations per sites of this class. For classes of cytosines that have few sites in the genome and/or few observed mutations, the uncertainty on this estimated relative mutation frequency becomes large. Therefore, we estimated a 95% confidence interval on the ratio, by first approximating the standard deviation (SD) of the ratio by propagating an assumed square-root uncertainty in the integer counts, using the formula SD = (n/N) × [(n½/n)2 + (N½/N)2]½, and then estimating the 95% confidence interval as (n/N) ± 1.96 × SD. Finally, we normalized this ratio (and confidence interval) to a relative mutation frequency, by dividing the sample cohort's baseline rate of mutations at all TCA trinucleotides in the genome (the most favored site for APOBEC mutations). The bar plots in **Figures. 6A, B, D, E** and **Supplementary Figure 9A** show this estimated relative mutation frequency, with the estimated 95% confidence interval shown as error bars. This analysis is part of the analyze_mutations function of ApoHP.

Mutation calls from TCGA whole-exome sequencing (WXS) were obtained from the TCGA Unified Ensemble MC3 Call Set (5), the public, open-access dataset of somatic mutation calls produced by the MC3 calling effort (Multi-Center Mutation Calling in Multiple Cancers), downloaded from the following link: [http://www.synapse.org/#!Synapse:syn7214402/wiki/405297] (The results here are in whole or part based upon data generated by the TCGA Research Network: [http://cancergenome.nih.gov/] as outlined in the TCGA publications guidelines [http://cancergenome.nih.gov/publications/publicationguidelines]). Following the filtering procedure that was used for the PanCanAtlas project, the MC3 dataset was filtered to include only PASS variants, which removes patients that were subjected to whole-genome amplification (WGA), as well as the acute myeloid leukemia (LAML) cohort. This yielded a final cohort of 9023 patients covering 32 tumor types. Mutation calls from whole-genome sequencing (WGS) from TCGA and other projects were obtained from the International Cancer Genome Consortium (ICGC) Pan-Cancer Analysis of Whole Genomes (PCAWG) project. Mutation calls were downloaded from the ICGC Portal (https://dcc.icgc.org/api/v1/download?fn=/PCAWG/consensus_snv_indel/final_consensus_passonly.snv_mnv_indel.icgc.public.maf.gz and https://dcc.icgc.org/api/v1/download?fn=/PCAWG/consensus_snv_indel/final_consensus_snv_indel_passonly_icgc.public.tgz). Note that controlled tier access credentials are required from the ICGC and TCGA projects as described on the ICGC PCAWG site [http://docs.icgc.org/pcawg/data/]. Additional WGS data were obtained from published projects from the following links: [ftp://ftp.sanger.ac.uk/pub/cancer/AlexandrovEtAl/somatic_mutation_data] and [ftp://ftp.sanger.ac.uk/pub/cancer/Nik-ZainalEtAl-560BreastGenomes/Caveman_560_20Nov14_clean.txt]. Identifying and removing duplicate patients, restricting to somatic single-nucleotide variants (SSNVs), and excluding patients with

fewer than 500 SSNVs in the genome yielded a final WGS dataset comprising 2800 unique patients spanning 35 tumor types.

SUPPLEMENTARY REFERENCES

1. Alexandrov,L.B., Nik-Zainal,S., Wedge,D.C., Aparicio,S.A.J.R., Behjati,S., Biankin,A. V., Bignell,G.R., Bolli,N., Borg,A., Børresen-Dale,A.-L., *et al.* (2013) Signatures of mutational processes in human cancer. *Nature*, **500**, 415–421.
2. Nik-Zainal,S., Davies,H., Staaf,J., Ramakrishna,M., Glodzik,D., Zou,X., Martincorena,I., Alexandrov,L.B., Martin,S., Wedge,D.C., *et al.* (2016) Landscape of somatic mutations in 560 breast cancer whole-genome sequences. *Nature*, **534**, 47–54.
3. Law,E.K., Levin-Klein,R., Jarvis,M.C., Kim,H., Argyris,P.P., Carpenter,M.A., Starrett,G.J., Temiz,N.A., Larson,L.K., Durfee,C., *et al.* (2020) APOBEC3A catalyzes mutation and drives carcinogenesis in vivo. *J Exp Med*, **217**.
4. Durfee,C., Temiz,N.A., Levin-Klein,R., Argyris,P.P., Alsøe,L., Carracedo,S., Alonso de la Vega,A., Proehl,J., Holzhauer,A.M., Seeman,Z.J., *et al.* (2023) Human APOBEC3B promotes tumor development in vivo including signature mutations and metastases. *Cell Rep Med*, **4**.
5. Ellrott,K., Bailey,M.H., Saksena,G., Covington,K.R., Kandoth,C., Stewart,C., Hess,J., Ma,S., Chiotti,K.E., McLellan,M., *et al.* (2018) Scalable Open Science Approach for Mutation Calling of Tumor Exomes Using Multiple Genomic Pipelines. *Cell Syst*, **6**, 271-281.e7.
