# Supplementary material for: Mesoscale DNA features impact APOBEC3A and APOBEC3B deaminase activity and shape tumor mutational landscapes
Source: Nat Commun. 2024 Mar 18;15:2370. doi: 10.1038/s41467-024-45909-5 (PMC10948877; doi:10.1038/s41467-024-45909-5)
Supplement: Supplementary file 3 — Description of Additional Supplementary Files [file 41467_2024_45909_MOESM3_ESM.pdf]

Description of Additional Supplementary Files

File Name: Supplementary Data 1

Number of mutations identified in mouse or human tumors caused by A3A or A3B in the indicated types of DNA structures.
