# Supplementary material for: Mesoscale DNA features impact APOBEC3A and APOBEC3B deaminase activity and shape tumor mutational landscapes
Source: Nat Commun. 2024 Mar 18;15:2370. doi: 10.1038/s41467-024-45909-5 (PMC10948877; doi:10.1038/s41467-024-45909-5)
Supplement: Supplementary file 5 — Reporting Summary [file 41467_2024_45909_MOESM5_ESM.pdf]

nature portfolio

Corresponding author(s): Rémi Buisson

Last updated by author(s): Dec 18, 2023

Reporting Summary

Nature Portfolio wishes to improve the reproducibility of the work that we publish. This form provides structure for consistency and transparency in reporting. For further information on Nature Portfolio policies, see our Editorial Policies and the Editorial Policy Checklist.

Statistics

For all statistical analyses, confirm that the following items are present in the figure legend, table legend, main text, or Methods section.

n/a	Confirmed	
☐	☒	The exact sample size (*n*) for each experimental group/condition, given as a discrete number and unit of measurement
☐	☒	A statement on whether measurements were taken from distinct samples or whether the same sample was measured repeatedly
☐	☒	The statistical test(s) used AND whether they are one- or two-sided<br>*Only common tests should be described solely by name; describe more complex techniques in the Methods section.*
☒	☐	A description of all covariates tested
☒	☐	A description of any assumptions or corrections, such as tests of normality and adjustment for multiple comparisons
☐	☒	A full description of the statistical parameters including central tendency (e.g. means) or other basic estimates (e.g. regression coefficient) AND variation (e.g. standard deviation) or associated estimates of uncertainty (e.g. confidence intervals)
☐	☒	For null hypothesis testing, the test statistic (e.g. *F*, *t*, *r*) with confidence intervals, effect sizes, degrees of freedom and *P* value noted<br>*Give P values as exact values whenever suitable.*
☒	☐	For Bayesian analysis, information on the choice of priors and Markov chain Monte Carlo settings
☒	☐	For hierarchical and complex designs, identification of the appropriate level for tests and full reporting of outcomes
☐	☒	Estimates of effect sizes (e.g. Cohen's *d*, Pearson's *r*), indicating how they were calculated

Our web collection on statistics for biologists contains articles on many of the points above.

Software and code

Policy information about availability of computer code

Data collection	Gel images were acquired using a Chemidoc MP (BioRad) and analysed with ImageLab 6.1 .
Data analysis	Gel images were quantified with ImageLab 6.1 (Biorad).<br>Graphs were generated using Prism 9 (GraphPad).<br>Source code and executable software tool ApoHP are available at http://github.com/alangenb/ApoHP

For manuscripts utilizing custom algorithms or software that are central to the research but not yet described in published literature, software must be made available to editors and reviewers. We strongly encourage code deposition in a community repository (e.g. GitHub). See the Nature Portfolio guidelines for submitting code & software for further information.

Data

Policy information about availability of data

All manuscripts must include a data availability statement. This statement should provide the following information, where applicable:

- Accession codes, unique identifiers, or web links for publicly available datasets
- A description of any restrictions on data availability
- For clinical datasets or third party data, please ensure that the statement adheres to our policy

Mutation calls from TCGA whole-exome sequencing (WXS) were obtained from the TCGA Unified Ensemble MC3 Call Set (91), the public, open-access dataset of somatic mutation calls produced by the MC3 calling effort (Multi-Center Mutation Calling in Multiple Cancers), downloaded from the following link: [http://

www.synapse.org/#!Synapse:syn7214402/wiki/405297] (The results here are in whole or part based upon data generated by the TCGA Research Network: [http://cancergenome.nih.gov/] as outlined in the TCGA publications guidelines [http://cancergenome.nih.gov/publications/publicationguidelines]). Following the filtering procedure that was used for the PanCanAtlas project, the MC3 dataset was filtered to include only PASS variants, which removes patients that were subjected to whole-genome amplification (WGA), as well as the acute myeloid leukemia (LAML) cohort. This yielded a final cohort of 9023 patients covering 32 tumor types. Mutation calls from whole-genome sequencing (WGS) from TCGA and other projects were obtained from the International Cancer Genome Consortium (ICGC) Pan-Cancer Analysis of Whole Genomes (PCAWG) project. Mutation calls were downloaded from the ICGC Portal (https://dcc.icgc.org/api/v1/download?fn=/PCAWG/consensus_snv_indel/final_consensus_passonly.snv_mnv_indel.icgc.public.maf.gz and https://dcc.icgc.org/api/v1/download?fn=/PCAWG/consensus_snv_indel/final_consensus_snv_indel_passonly_icgc.public.tgz). Note that controlled tier access credentials are required from the ICGC and TCGA projects as described on the ICGC PCAWG site [http://docs.icgc.org/pcawg/data/]. Additional WGS data were obtained from published projects1,22 from the following links: [ftp://ftp.sanger.ac.uk/pub/cancer/AlexandrovEtAl/somatic_mutation_data] and [ftp://ftp.sanger.ac.uk/pub/cancer/Nik-ZainalEtAl-560BreastGenomes/Caveman_560_20Nov14_clean.txt]. Identifying and removing duplicate patients, restricting to somatic single-nucleotide variants (SSNVs), and excluding patients with fewer than 500 SSNVs in the genome yielded a final WGS dataset comprising 2800 unique patients spanning 35 tumor types.

Sequencing reads from mouse tumors are available at the NCBI SRA under BioProject ID: PRJNA927047 and PRJNA655491. Sequencing results generated from Oligo seq experiments are available at the NCBI SRA under BioProject ID: PRJNA1010353.

Research involving human participants, their data, or biological material

Policy information about studies with human participants or human data. See also policy information about sex, gender (identity/presentation), and sexual orientation and race, ethnicity and racism.

Reporting on sex and gender	not applicable
Reporting on race, ethnicity, or other socially relevant groupings	not applicable
Population characteristics	not applicable
Recruitment	not applicable
Ethics oversight	not applicable

Note that full information on the approval of the study protocol must also be provided in the manuscript.

Field-specific reporting

Please select the one below that is the best fit for your research. If you are not sure, read the appropriate sections before making your selection.

☒ Life sciences ☐ Behavioural & social sciences ☐ Ecological, evolutionary & environmental sciences

For a reference copy of the document with all sections, see nature.com/documents/nr-reporting-summary-flat.pdf

Life sciences study design

All studies must disclose on these points even when the disclosure is negative.

Sample size	Sample sizes were chosen according to accepted standards in the field. Sample size was not pre-determined using statistics tolls. As indicated in the figure legends, minimal size of analyzed biological samples was "3". Statistical analysis (as described in respective figure legends) was used to calculate statistical significance of obtained results. The individual p-values are indicated in figures or in figure legends.
Data exclusions	No data were excluded.
Replication	All experiments have been repeated in multiple successfully independent experiments (3 times or more).
Randomization	We had a limited number of biological samples. The analysis was self-normalized to the sample, so randomization of samples would not be a relevant method.
Blinding	As the analysis required comparisons against a known controls and knockdown targets were selected for their likely relevance to the biological pathway, blinding would not provide much reduction of potential bias in the analysis. However, performance and analyses of experiments were independently conducted by co-authors.

Reporting for specific materials, systems and methods

We require information from authors about some types of materials, experimental systems and methods used in many studies. Here, indicate whether each material, system or method listed is relevant to your study. If you are not sure if a list item applies to your research, read the appropriate section before selecting a response.

Materials & experimental systems

n/a	Involved in the study
☐	☒ Antibodies
☐	☒ Eukaryotic cell lines
☒	☐ Palaeontology and archaeology
☒	☐ Animals and other organisms
☒	☐ Clinical data
☒	☐ Dual use research of concern
☒	☐ Plants

Methods

n/a	Involved in the study
☒	☐ ChIP-seq
☒	☐ Flow cytometry
☒	☐ MRI-based neuroimaging

Antibodies

Antibodies used

The antibodies used in this study were: GAPDH polyclonal antibody (EMD Millipore #ABS16), Flag polyclonal antibody (Sigma-Aldrich, M2, #F7425), Flag monoclonal antibody (Sigma-Aldrich #F1804), UNG polyclonal antibody (Novus biologicals # NBP1-49985), RPA32 monoclonal antibody (Invitrogen, 9H8, #MA1-26418), Vinculin monoclonal antibody (Sigma-Aldrich, hVin-1, #V9264), and APOBEC3B monoclonal antibody (5210-87-13; NIH-ARP #12398).

Validation

Antibodies specificity against UNG and APOBEC3B were confirmed in this manuscript using knockout cell or knockdown lines for the corresponding protein target. GAPDH and Flag antibodies were validated by the manufacturer for western blots and referenced in other papers that are included in the manufacturer website. RPA were previously used in many other publications (e.g., Buisson et al. Molecular Cell, 2015)

Eukaryotic cell lines

Policy information about cell lines and Sex and Gender in Research

Cell line source(s)

U2OS and HEK293T cell lines were purchased from either ATCC or Sigma-Aldrich. Enginering cell lines were derived from U2OS cell line as described in the method section.

Authentication

All cell lines were obtained from commercial repositories (ATTC, Sigma Aldrish). Upon receipt, the cell lines were expanded and frozen stocks were created. For the experiments described in this article, cell lines were not continuously kept in culture for more than 3 months. These cell lines were not authenticated

Mycoplasma contamination

All cell lines were tested repetitively for Mycoplasma contamination and all cell lines were tested negative for Mycoplasma

Commonly misidentified lines
(See ICLAC register)

No commonly misidentified cell lines were used in the study
